# Combined Effects of Nonylphenol and Bisphenol A on the Human Prostate Epithelial Cell Line RWPE-1

**DOI:** 10.3390/ijerph120404141

**Published:** 2015-04-14

**Authors:** Weidong Gan, Ming Zhou, Zou Xiang, Xiaodong Han, Dongmei Li

**Affiliations:** 1 Department of Urology, Nanjing Drum Tower Hospital, The Affiliated Hospital of Medical School, Nanjing University, Nanjing 210008, China; E-Mail: gwd@nju.edu.cn; 2 Immunology and Reproductive Biology Laboratory & State Key Laboratory of Analytical Chemistry for Life Science, Medical School, Nanjing University, Nanjing 210093, China; E-Mail: hanxd@nju.edu.cn; 3 Jiangsu Key Laboratory of Molecular Medicine, Nanjing University, Nanjing 210093, China; 4 Department of Urology, Yangzhou First People’s Hospital, Yangzhou 225000, China; E-Mail: nip_war@sina.com; 5 Department of Microbiology and Immunology, Mucosal Immunobiology and Vaccine Research Center, Institute of Biomedicine, University of Gothenburg, Gothenburg 405 30, Sweden; E-Mail: swesala@hotmail.com

**Keywords:** nonylphenol (NP), bisphenol A (BPA), combined effect, RWPE-1, Loewe additivity (LA) model, Bliss independence (BI) model

## Abstract

The xenoestrogens nonylphenol (NP) and bisphenol A (BPA) are regarded as endocrine disrupting chemicals (EDCs) which have widespread occurrence in our daily life. In the present study, the purpose was to analyze the combined effects of NP and BPA on the human prostate epithelial cell line RWPE-1 using two mathematical models based on the Loewe additivity (LA) theory and the Bliss independence (BI) theory. RWPE-1 cells were treated with NP (0.01–100 µM) and BPA (1–5000 µM) in either a single or a combined format. A cell viability assay and lactate dehydrogenase (LDH) leakage rate assay were employed as endpoints. As predicted by the two models and based on the cell viability assay, significant synergism between NP and BPA were observed. However, based on the LDH assay, the trends were reversed. Given that environmental contaminants are frequently encountered simultaneously, these data indicated that there were potential interactions between NP and BPA, and the combined effects of the chemical mixture might be stronger than the additive values of individual chemicals combined, which should be taken into consideration for the risk assessment of EDCs.

## 1. Introduction

It is known that all living organisms are constantly and unavoidably exposed to a variety of synthetic chemicals through food, air, water and dermal contact. Nonyl phenol (NP) and bisphenol A (BPA), two well-known man-made chemicals, also referred to as endocrine disruptor chemicals (EDCs), are persistent environmental contaminants that are priority substances under the European Union Water Framework Directive [1]. NP is a primary degradation product of nonylphenol ethoxylate (NPEO), a major group of multipurpose nonionic surface active agents which are widely used as pesticide emulsifiers, defoaming agents, softening agents, dyeing auxiliaries, and finishing agents in the textile industry. BPA has been widely used for its cross-linking properties in the manufacture of polycarbonate plastics and epoxy resins. Because of incomplete polymerization and degradation of the polymers, BPA has been found to easily leach in microgram amounts from polycarbonate plastics and epoxy resins into food and water supplies [2]. Exposure to NP and BPA is nearly ubiquitous, and urinary analysis revealed that NP was detected in 51% of the samples from a population of 394 adults and BPA is detected in >93% of the population in the United States [3,4] The human body is exposed to concentration of 7.5 μg/day of NP and 10 μg/kg/day BPA in the way which they leach from the lining of plastic packages, cans and baby bottles, and pipe walls [3,5].

NP and BPA are both proposed to have a myriad of effects on human health, especially on the reproductive system [6,7]. Our previous studies have indicated NP could cause adverse reproduction effects in male rats [8,9]. BPA also could effect on the reproduction and development of human and laboratory animals in many ways, such as fertility, male sexual function, sperm quality, sex hormone concentrations, birth weight, and childhood behavior/neurodevelopment, and some diseases or symptoms, such as reduced sperm quality, polycystic ovary syndrome, breast cancer, endometrial disorders, miscarriage, and male genital abnormalities, were found to be associated with BPA exposure [6].

Both NP and BPA are not only considered to mimic the effect of estrogen [10,11], but they could also exert toxicity by disrupting the cell survival and death pathways. In addition, health risk assessments of exposure to chemicals are based on data from studies on the individual substances. However, humans are always simultaneously exposed to a large number of chemicals that potentially possess a number of similar or different toxic effects which may produce unforeseen effects. Therefore the aspect of combined actions of chemicals needs to be addressed to a greater extent in the risk assessment process.

The prostate is an important organ of the male reproductive system which secretes a slightly alkaline fluid that in humans usually constitutes roughly 30% of the volume of the semen [12]. Prostate diseases, such as prostatitis, enlarged prostate and prostate cancer, become very common with age. It was shown that there is a correlation between prostate problems and NP or BPA exposure [13,14,15]. In the present study, the purpose was to investigate the combined effects of NP and BPA and to elucidate the mode of interaction between NP and BPA in the nontumorigenic human prostate epithelial cell line RWPE-1. We used two separate quantitative assays to measure cell viability: the CCK-8 mitochrondrial dehydrogenase assay and the lactate dehydrogenase (LDH) membrane leakage assay. A nonlinear regression model was used to analyze the dose-response relationships of the individual compounds, and the Loewe additivity (LA) model and the Bliss independence (BI) model were applied to analyzing the combined effects of a mixture of NP and BPA.

## 2. Results

### 2.1. Concentration-response Analysis of Nonylphenol (NP) and Bisphenol A (BPA) Alone or in Combination in the Cell Viability Assay

**Figure 1:**
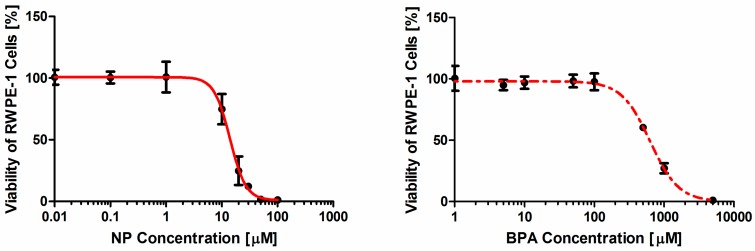
Concentration-response curves for NP and BPA on the cell viability of RWPE-1 cell regression from the Hill function. Experimental data are shown for at least three tested donors (each in sextuplicate).

The inhibitory effect of NP and BPA on the viability of RWPE-1 cells was dose-dependent. The concentration-response curves of NP and BPA are both sigmoid, but have different effectiveness with similar slopes (Figure 1). The parameters satisfying the Hill-function are summarized in Table 1, including slopes, goodness-of-fit criteria and the calculated EC_50_ values. The regression-based EC_50_ values of NP and BPA varied substantially. NP was much more effective with an EC_50_ of 15 μM than BPA with an EC_50_ of 610.27 μM. In addition, the two contaminants had similar slopes which were 2.68 for NP and 2.10 for BPA. However, the EC_50_ values of NP and BPA in the combination were 7.44 and 297.68 μM, respectively (Table 1).

**Table 1:** The concentration-response curve of the viability assay satisfying the Hill function of the chemicals NP and BPA alone or in combination.

Hill-Function Parameters (α ± 95CI)	Viability Assay		
	NP	BPA	Combined
Slope (*p*) ^a^	−2.68 ± 0.24	−2.10 ± 0.23	−3.217 ± 0.2161
EC_50_ (μM) ^b^	15.00	610.27	7.44 (NP); 297.68 (BPA)
Vmax (%) ^c^	1.008 ± 0.018	0.981 ± 0.01	0.9956 ± 0.014
*R*^2 d^	0.9726	0.9796	0.9781
Chi^2^/DoF ^e^	0.2543	0.1309	0.3384

^a^ Slope parameter of the Hill function; ^b^ Median effect concentration, *i.e.*, concentration yielding 50% of the maximal effect produced by the chemicals; ^c^ Maximal effect, expressed as corrected absorbance readings; ^d,e^ Goodness of fit criteria of the Hill function.

### 2.2. Concentration-response Analysis of Nonylphenol (NP) and Bisphenol A (BPA) alone or in Combination in the Lactate Dehydrogenase (LDH) Leakage Rate Assay

**Figure 2:**
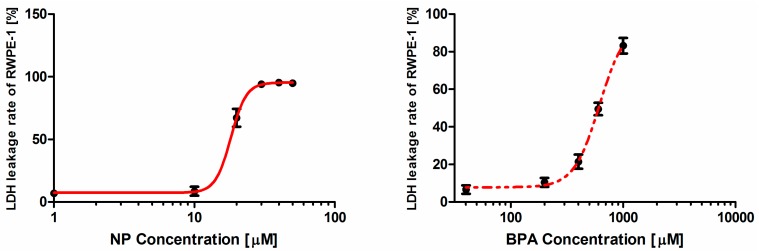
Concentration-response curves for nonylphenol (NP) and bisphenol A (BPA) on the lactate dehydrogenase (LDH) leakage rate of RWPE-1 cell regression from the Hill function. Experimental data are shown for at least three tested donors (each in sextuplicate).

The LDH leakage rate of RWPE-1cells increased monotonically with increasing concentrations of NP and BPA (Figure 2). The parameters satisfying the Hill-function are summarized in Table 2. The calculated EC_50_ values of NP and BPA were 18.18 μM and 616.3 μM, respectively. In addition, the EC_50_ values of NP and BPA in the combination was 22.18 μM and 776.3 μM, respectively. However, in this assay the two contaminants had different slopes which were 7.92 for NP and 4.69 for BPA (Table 2).

**Table 2:** The concentration-response curve of lactate dehydrogenase (LDH) leakage rate assay satisfying the Hill function of the chemicals nonylphenol (NP) and bisphenol A (BPA) alone or in combination.

Hill-Function Parameters (α ± 95%CI)	LDH leakage Assay		
	NP	BPA	Combined
Slope (*p*) ^a^	7.92 ± 2.13	4.69 ± 0.48	2.94 ± 0.2129
EC50 (μM) ^b^	18.18	616.3	22.18 (NP); 776.3 (BPA)
Vmax (%) ^c^	0.9535 ± 0.010	1.01 ± 0.04	0.9956 ± 0.024
*R*^2 d^	0.9940	0.9874	0.9907
Chi^2^/DoF ^e^	0.3306	0.4522	0.6615

^a^ Slope parameter of the Hill function; ^b^ Median effect concentration, *i.e.*, concentration yielding 50% of the maximal effect produced by the chemicals; ^c^ Maximal effect, expressed as corrected absorbance readings; ^d,e^ Goodness of fit criteria of the Hill function.

### 2.3. The Analysis by the Two Models for Determining the Combined Effects of Nonylphenol (NP) and Bisphenol A (BPA) on Cell Viability

To evaluate which type of combined effects was provoked by the mixture, the observed values caused by the mixture were compared with the predicted effects based on the concept of concentration additivity and on the alternative concept of response additivity.

**Figure 3:**
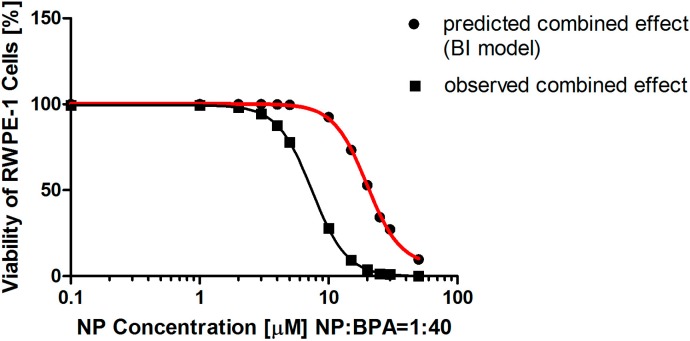
Comparison of observed and predicted cell viability caused by a combination of nonylphenol (NP) and bisphenol A (BPA). The predicted concentration-response curve of the mixture matched the data calculated from the Bliss independence (BI) models. Components were mixed in a fixed ratio based on their individual EC50 values. The two curves both satisfied the Hill function.

The concentration-response curves of NP or BPA alone in the cell viability assay have similar slopes. Therefore, LCI and its 95% confidence interval were calculated in the Loewe additivity (LA) model using the experimental data to determine the nature of the combined effect of the mixture of NP and BPA. As shown in Table 3, LCI and its 95% confidence interval were greater than 1 when the viability following the treatment of the mixture was higher than 60%, which suggested that a probable antagonistic effect existed between NP and BPA on inhibiting the viability of RWPE-1 cells at the concentrations corresponding to viabilities higher than 60%. In contrast, a probable synergistic effect between NP and BPA was observed on the viability of RWPE-1 cells at the concentrations corresponding to viabilities lower than 60% when the concentrations of NP and BPA were below 6.54 μM and 261.53 μM, respectively. With increasing concentration, a synergistic effect became more evident (Table 3). When using the BI model, a synergistic effect was predicted (Table 4). The difference in the prediction by the LA model and the BI model (Table 4 and Figure 3) was restricted to the relative low dose combinations of NP and BPA, such as NP at 3.71–6.54 μM in the combination, where the combined effects were antagonistic from the LA model instead of being synergistic from the BI model, but with increasing dose, there was no difference between the two models and the mixture provoked a synergistic effect on the cytotoxicity to RWPE-1 cells.

**Table 3:** Effect of single and combined nonylphenol (NP) and bisphenol A (BPA) on viability of RWPE-1 cells and the LCI derived from the Loewe additivity (LA) model.

Dose (μM)	Series of Viability Effects (%)								
	E90	E80	E70	E60	E50	E40	E30	E10	E5
D_NP_^a^	6.8	9.07	11.04	12.99	15.09	17.54	20.67	34.17	45.1492
D_BPA_^b^	193.66	300.58	395.02	491.47	599 00	728.87	901.56	1719.74	2456.00
d_NP_^c^	3.71	4.81	5.69	6.54	7.42	8.42	9.67	14.71	18.56
d_BPA_^d^	148.27	192.13	227.70	261.53	296.86	336.89	386.62	588.45	742.37
LCI ^e^	1.31	1.17	1.09	1.04	0.99	0.94	0.90	0.77	0.71
±95% Confidence interval	1.18~1.34	1.11~1.18	1.06~1.1	1.01~1.07	0.97~0.99	0.93~0.95.	0.84~0.94	0.66~0.88	0.58~0.85
Combined effect	antagonism	antagonism	antagonism	antagonism	synergism	synergism	synergism	synergism	synergism

^a,b^ The iso-effective concentrations of NP and BPA when acting alone; ^c,d^ Concentrations of the chemicals NP and BPA in the combination which elicit a certain effect; ^e^ Loewe combination Index.

**Table 4:** Predicted and observed effects of the mixture of nonylphenol (NP) and bisphenol A (BPA) on viability of RWPE-1 cells from the Bliss independence (BI) model.

d_NP_ (μM) ^a^	5.00	10.00	15.00	20.00	25.00	30.00	50.00
d_BPA_ (μM) ^b^	200.00	400.00	600.00	800.00	1100.00	1200.00	2000.00
E_NP_^c^	0.97	0.74	0.45	0.25	0.97	0.74	0.45
E_BPA_^d^	0.90	0.71	0.52	0.37	0.90	0.71	0.52
Predicted effect ^e^	1.00	0.92	0.75	0.56	0.38	0.30	0.11
Observed effect ^f^	0.78	0.28	0.09	0.04	0.02	0.01	0.09
*q* value	0.78	0.30	0.13	0.07	0.05	0.04	0.02
Combined effect	synergism	synergism	synergism	synergism	synergism	synergism	synergism

^a,b^ Concentrations of the chemicals NP and BPA in the combination which elicit a certain effect; ^c,d^ The effects of NP and BPA when acting alone; ^e^ The predicted effect of the non-interactive combination of the chemicals NP and BPA; ^f^ The observed combined effect of the chemicals NP and BPA.

### 2.4. The Analysis by the Two Models for Determining the Combined Effects of Nonylphenol (NP) and Bisphenol A (BPA) on Lactate Dehydrogenase (LDH) Leakage Rate

**Figure 4:**
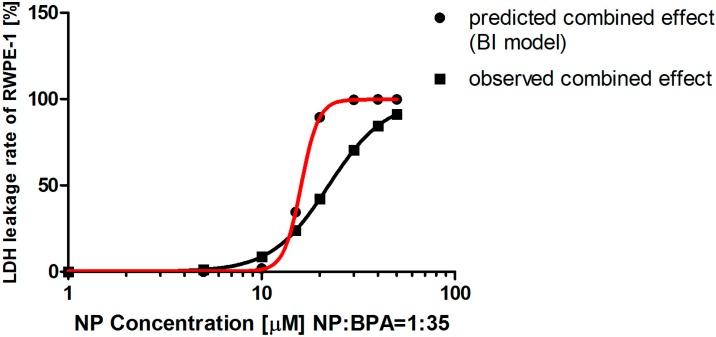
Comparison of observed and predicted lactate dehydrogenase (LDH) leakage rate caused by a combination of nonylphenol (NP) and bisphenol A (BPA). The predicted concentration-response curve of the mixture matched the data calculated from the Bliss independence (BI) models. Components were mixed in a fixed ratio based on their individual EC_50_ values. The two curves both satisfied the Hill function.

In the LDH assay, we also observed discrepancies in the prediction by the two models, especially at relatively low doses of NP and BPA in combination. Because the concentration-response curves of NP or BPA alone in the cell viability assay have different slopes, Equation (4) was applied in the LA model to calculate the combination index (CI) using the experimental data to determine the combined effect of the mixture of NP and BPA. As shown in Table 5, an antagonistic effect was observed in combination at the concentrations where the LDH leakage rate higher than 1% was induced in both LA model. However, there were certain discrepancies in the prediction by the two models. When the concentrations of NP and BPA in the combination were lower than 10 and 350 μM, respectively, the combined effect was a probable synergistic effect by the BI model (Table 6 and Figure 4). But, with increasing dose, there was no difference between the two models and the mixture consistently provoked an antagonistic effect on LDH leakage in RWPE-1 cells.

**Table 5:** Effect of single and combined nonylphenol (NP) and bisphenol A (BPA) on the lactate dehydrogenase (LDH) leakage rate of RWPE-1 cells and the combination index (CI) derived from the Loewe additivity (LA) model.

Dose (μM)	Series of LDH Leakage Rate (%)							
	E1	E10	E40	E50	E60	E70	E80	E90
D_NP_^a^	10.24	13.87	17.45	18.41	19.44	20.67	22.39	25.96
D_BPA_^b^	230.77	384.79	563.25	613.70	668.45	733.24	819.78	964.97
d_NP_^c^	4.65	10.52	19.37	22.25	25.56	29.74	35.81	47.55
d_BPA_^d^	162.89	368.28	677.99	778.64	894.45	1040.82	1253.42	1664.37
CI	1.48	2.44	3.65	4.01	4.41	4.90	5.57	6.72
Combined effect	antaonism	antagonism	antagonism	antagonism	antagonism	antagonism	antagonism	antagonism

^a,b^ The iso-effective concentrations of NP and BPA when acting alone; ^c,d^ Concentrations of the chemicals NP and BPA in the combination which elicit a certain effect; ^e^ Combination Index.

**Table 6:** Predicted and observed effect of the mixture of nonylphenol (NP) and bisphenol A (BPA) on the lactate dehydrogenase (LDH) leakage rate of RWPE-1 cells from the Bliss independence (BI) model.

d_NP_ (μM) ^a^	1.00	5.00	10.00	15.00	20.00	30.00
d_BPA_ (μM) ^b^	35.00	175.00	350.00	525.00	700.00	1050.00
E_NP_^c^	0.00	0.00	0.01	0.17	0.65	0.94
E_BPA_^d^	0.00	0.00	0.07	0.32	0.65	0.93
Predicted effect ^e^	0.00	0.00	0.07	0.44	0.88	1.00
Observed effect ^f^	0.00	0.01	0.09	0.24	0.42	0.71
*q* value	75.25	4.42	1.18	0.55	0.48	0.71
Combined effect	synergism	synergism	synergism	antagonism	antagonism	antagonism

^a,b^ Concentrations of the chemicals NP and BPA in the combination which elicit a certain effect; ^c,d^ The effects of NP and BPA when acting alone; ^e^ The predicted effect of the non-interactive combination of the chemicals NP and BPA; ^f^ The observed combined effect of the chemicals NP and BPA.

## 3. Discussion

With the widespread occurrence of NP and BPA, both them are frequently detected in the human body [16]. Vandenberg *et al.* [17] reviewed the remarkable situation of the presence of BPA in human fluids and tissues in the majority of individuals in many developed countries, such as serum, blood, plasma, pregnancy-associated fluids, breast milk, urine, semen, and follicular fluid.

Our study showed that each of the two contaminants induced toxic effect on RWPE-1 cells in a dose-dependence manner in the micromolar range. The toxicity ranking for the individual contaminant is NP > BPA, with NP being at least 30 times more active in the assay than BPA. The viability assay and LDH leakage rate assay were chosen as two endpoints for a sensible testing approach because both assays provided reproducible quantitative data with relatively few variations and could detect small changes among different dose-groups, which makes the assessment more credible and precise.

The LA model and BI model are widely used mathematical models for analyzing drug interactions *in vitro* [18]. They have different theoretical basis. The LA model which is derived from the concept of concentration addition (CA) [19] focuses on comparison in concentration and is applied to judging the interaction between chemicals by comparing the actual concentration in the mixture and the theoretical concentration without interaction between chemicals. In the case of CA, the contribution to the final toxicity of the mixture depends on the relative potency of the components assumed to be constant throughout all dose levels. In principle no threshold exists for CA which means the LA model can be applied to analyzing combined effects of chemicals at low environmental exposure levels [20]. On the basis of probability theory, the BI model which is derived from the concept of response addition (RA) is applied to judging the interaction between chemicals through comparing the observed effect of the combined chemicals and the predicted effect without interaction between the chemicals [18]. However, the BI model cannot be used at levels at which the individual compounds elicit no response [21]. Thus, Faust and his colleagues demonstrated that the LA model yielded more accurate predictions with agents that interact with the same molecular site [19].

In the present study, we found that the combined effects of NP and BPA are correlated to the doses adopted in the same test endpoint, as well as, even at the same doses in the combination, different combined effects were observed in the different test endpoint. In the cell viability assay, both the LA and BI model predicted a synergistic mixture effect of the two chemicals, but the LA model more accurately predicted an antagonistic combined effect at a low-level dose. Since the mixture effect calculated by the BI model deviated from the observed effect, this suggested that this model was not suitable to describe an accurate combined effect in the viability assay. For the LDH leakage assay, the LA model predicted an antagonistic combined effect, but in the BI model, at low-level doses, the two chemicals act synergistically while at high-level doses the two chemicals act antagonistically. These results are not consistent with previous report in which no significant interactions occur between NP and BPA when the effects of mixture of NP and BPA was investigated using plasma vitellogenin induction in male adult Japanese medaka (*Oryzias latipes*) as the endpoint [22]. In that study, however, factorial analysis was used instead of the mathematical model employed in our present study. Taken together, these results indicated that the multiple joint actions of NP and BPA might be related to the complexity of their individual mechanisms of action.

In view of the different end points, the combined effect of NP and BPA obtained in the two experiments were not consistent, presumably due to the fact that different interactions occur on different targets. Be it synergism or antagonism, competitive binding is most likely one of the mechanisms for the interpretation of the interaction between two chemicals. For example, it was reported that both 4-n-nonylphenol (nNP) and BPA could inhibit aromatase activity and were agonists and antagonists of ER and androgen receptor (AR), respectively, and the aryl hydrocarbon receptor (AhR) activity was inhibited with BPA (10–100 μM) [23]. Further, it is demonstrated that NP can exert an estrogen-like transcriptional activity on ER-positive breast cancer cells by binding to ER-α [24], and another study showed that BPA could bind to two ERs, ERα and ERβ, and be considered a selective ER modulator (SERM) that partially activates luciferase reporter in MCF-7 and HeLa cells. [25]. Moreover, other studies showed direct interaction between BPA and several other receptors including K-ras and the orphan nuclear estrogen related receptor (ERR) γ [26,27].

The combined effects we observed in the current study were investigated *in vitro*. By using two mathematical models, we compared the difference between observed value and predicted value, analyzed the combined effects of a mixture of NP and BPA, and found the interaction between these two chemicals. There is, however, limitation for predicting the effects of multiple exposures to low-dose environmental chemicals. Therefore, more sensitive methods and mathematical models should be developed for the risk assessment of EDCs.

## 4. Materials and Methods

### 4.1. Chemicals

Analytical grade (98%) 4-nonylphenol (NP) was purchased from Tokyo Kasei Kogyo Co. (Tokyo, Japan). BPA powder (99%) was purchased from Sigma-Aldrich (St. Louis, MO, USA). Stock solutions of NP and BPA were prepared in dimethyl sulfoxide (DMSO, Sigma-Aldrich) at final concentrations of 100 and 5000 mM, respectively. Serum-free keratinocyte medium (Keratinocyte-SFM) was purchased from Gibco (Thermo Fisher Scientific, Carlsbad, CA, USA). Penicillin, streptomycin sulfate, and trypsin were purchased from Sigma-Aldrich. Cell Counting Kit-8 (CCK-8) was obtained from Dojindo Lab (Kumamoto, Japan). The CytoTox-ONE™ Homogeneous Membrane Integrity Assay kit was purchased from Promega (Madison, WI, USA).

### 4.2. Culture of the Human Prostate Epithelial Cell Line RWPE-1

RWPE-1 was purchased from the American Type Culture Collection (ATCC) and seeded into culture dishes at a density of approximately 2 × 10^6^ cells /mL in 10 mL complete Keratinocyte-SFM medium containing 5 ng/mL recombinant epidermal growth factor (rEGF), 50 μg/mL bovine pituitary extract (BPE), 100 IU/mL penicillin, and 100 IU/mL streptomycin. Cells were incubated in a humidified atmosphere of 95% air, 5% CO2 at 37 °C. After culture for 5 days, 80%~90% RWPE-1 cells were attached to the bottom of the dishes and were digested with 0.25% trypsin for passaging.

### 4.3. Cell Viability Assay

Cell viability was determined by CCK-8. CCK-8 contains WST-8 which can be reduced to a hydrosoluble formazan dye by mitochondrial dehydrogenase in living cells. Briefly, RWPE-1 cells were trypsinized and transferred to 96-well culture plates at 200 μL per well at a density of 1 × 10^6^ cells/mL. After incubating for 24 h, cells were exposed to various concentrations of NP (0, 0.01, 0.1, 1, 10, 20, 30, 50, 100 μM) and/or BPA (0, 1, 5, 10, 50, 100, 500, 1,000, 5,000 μM). The concentrations of chemicals in the mixture were determined by the EC50s of the individual NP and BPA. Next, cells were incubated in a humidified atmosphere of 95% air, 5% CO_2_ at 37 °C. After incubation for 24 h, 10 μL CCK-8 solutions were added to each well followed by a further incubation at 37 °C for 4 h. The absorbance was measured on an automated microplate reader (Bio-Rad, CA, USA) at 450 nm. This experiment was repeated at least three times in sextuplicate for NP and BPA either alone or in combination.

### 4.4. Lactate Dehydrogenase (LDH) Leakage Rate Assay

The plasma membrane integrity was determined by an extracellular LDH leakage assay using the CytoTox-ONE™ Homogeneous Membrane Integrity Assay kit (Promega) which is a fluorometric method for estimating the number of nonviable cells present in multiwell plates.

RWPE-1 cell were trypsinized and transferred to 96-well culture plates at 100 μL per well at a density of 1 × 10^6^ cells/mL. After incubating for 24 h, cells were exposed to various concentrations of NP (0, 0.01, 0.1, 1, 10, 20, 30, 50, 100 μM) or BPA (0, 1, 5, 10, 50, 100, 500, 1000, 5000 μM). The concentrations of chemicals in the mixture were determined by the EC50s of the individual NP and BPA. Next, cells were incubated in a humidified atmosphere of 95% air, 5% CO_2_ at 37 °C for another 24 h. Then, according to protocols of kit, LDH release assay was performed. The LDH activity both in culture supernatants (*S*) and in the cells (*C*) after cell lysis was measured. Fluorescence was measured using excitation/emission wavelengths at 560/590 nm. The experiment was repeated at least three times in sextuplicate using different batches of RWPE-1. The percentage of LDH leakage was calculated as follows:

Leakage rate (%) = [S-blank] ÷ [(S-blank + C-blank)] × 100%
(1)

### 4.5. Chemical Combination and Data Analysis

#### 4.5.1. The Modeling for the Single Effect

In order to predict a credible interaction between NP and BPA, it is necessary to determine the individual effects of NP or BPA on RWPE-1 in advance. The data from the CCK-8 assay and the LDH leakage assay were quantitative. Therefore, the predicted concentration-response curve could be obtained by a nonweighted nonlinear regression analysis using the mathematical software Origin 7.5 (Origin-Lab, Northampton, MA, USA). The Hill model is best fitted to analyze biometrically the concentration-response relationships of the individual substances [28]. Goodness-of-fit criteria include the 95% confidence interval [19] of the fitted parameters, the coefficient of determination (*R*^2^), and the Chi-square per degree of freedom (Chi^2^/DoF). The Hill-function we used in the present study is:

(2) E(%)=Emax1+(xEC50)−P

where *E*_max_ denotes the maximal effect observed in the absence of the toxicant, *p* is the slope, *E* is the effect and *x* is the concentration of the chemical. *EC*_50_, half maximal effective concentration, refers to the concentration of a drug, antibody or toxicant which induces a response halfway between the baseline and maximum after a specified exposure time. It could be suitable for analyzing the experimental data using the Hill function, maintaining *EC*_50_ and *p* as variable parameters.

#### 4.5.2. Dose Selection of the Mixture of Nonylphenol (NP) and Bisphenol A (BPA)

In addition to the untreated control cells and cells exposed to each chemical alone, concentrations of a mixture of NP and BPA was selected based on equieffective concentrations in both assays, which means that the ratio of the components was kept constant, while only the total concentration of the mixture was varying. Therefore, the concentration ratio of the mixture of NP and BPA was 1: 40 for the cell viability measurement (NP (μM) + BPA (μM) at 0.1 + 4, 1 + 40, 5 + 200, 10 + 400, 15 + 600, 20 + 8, 30 + 1.2, or 50 + 2000). The concentration ratio of the mixture of NP and BPA was 1:35 for the LDH leakage assay (NP (μM) + BPA (μM) at 1 + 35, 10 + 350, 20 + 700, 30 + 1050, 40 + 1400, or 50 + 1750).

### 4.6. The Modelling for the Mixture Effect

In the present study, two different models, the LA model and BI model, were both used to assess the combined effect of the two chemicals on RWPE-1 *in vitro*. The LA model is valid for mixtures where the components have similar sites and modes of action. By this model, the activities and effects are not changed if one component is partially replaced by an equipotent amount of another [20]. The main assumption of the BI model, however, is that two or more chemicals act independently from one another. In particular, if the criterion can be fulfilled, the models, and possibly also the sites of action of the compounds in the mixture, always differ.

#### 4.6.1. The Loewe Additivity (LA) Models

This model defines zero interaction between two chemicals and is described by the following equation [21]:

*d*_A_/*D*_A_ + *d*_B_/*D*_B_ = 1 (3)

where *d*_A_ and *d*_B_ are the concentrations of the chemicals A and B in the combination, respectively, for eliciting a certain effect, and *D*_A_ and *D*_B_ are the iso-effective concentrations of A and B, respectively, when acting alone. On the basis of Equation (2), the *Loewe combination index* (*LCI*) was introduced to assess the interaction of the mixed compounds:

*LCI* = *d*_A_/*D*_A_ + *d*_B_/*D*_B_ (4)

If the experimental product of this equation is equal to 1, namely *LCI* = 1, the combined effect is considered additivity; if *LCI* > 1, the combined effect is antagonism; while if *LCI* < 1, significant synergism is claimed.

With this equation, 95% confidence interval for the Loewe combination index was calculated to gauge the uncertainties of the interaction between the two chemicals in terms of the combination concentrations. Synergy is concluded if the *LCI* is less than 1 and its 95% confidence interval also falls below 1. Antagonism is concluded if the *LCI* is greater than 1 and its 95% confidence interval also lies above 1. Additivity is suggested when the 95% confidence interval is also equal to 1.

If the slope in one of the concentration-response curves is different from that in the other, the transformation for the equation [22] is:

*CI* = *d*_A_/*D*_A_ + *d*_B_/*D*_B_ + (*d*_A_ × *d*_B_)/(*D*_A_ × *D*_B_) (5)

where *CI* means the combination index. If *CI* = 1, the combined effect is considered additivity; if *CI* > 1, the combined effect is antagonism; while if *CI* < 1, significant synergism is claimed.

#### 4.6.2. The Bliss Independence (BI) Model

This model is described by the following equation:

*E*_AB_ = *E*_A_ + *E*_B_ ‒ *E*_A_* × E*_B_ (6)

where *E*_AB_ is the predicted effect of the non-interactive combination of the chemicals A and B, and *E*_A_ and *E*_B_ are the effects of A and B, respectively, when they act alone (0 < *E*_A_; *E*_B_ < 1). The difference between the measured combined effects and the predicted values at various concentrations reflects the interaction of the two chemicals.

On the basis of Equation (5), the *q* value was introduced to assess the interaction of mixed compounds [23]:

*q* = *E*_A+B_/*E*_AB_ = *E*_A+B_/(*E*_A_ + *E*_B_ − *E*_A_ × *E*_B_) (7)

where *E*_A+B_ is the observed effect of the combination of A and B.

When the slope in the concentration-response curve is positive and the *q* value is between 0.85~1.15, the combined effect is considered additive; if *q* > 1.15, the combined effect is synergistic; and if *q* > 20, significant synergism is claimed. While if *q* < 0.85, the combined effect is antagonistic; and if *q* < 0.55, significant antagonism is claimed.

When the slope in the concentration-response curve is negative and the *q* value is between 0.85~1.15, the combined effect is considered additivity; if *q* > 1.15, the combined effect is antagonism, and if *q* > 20, significant antagonism is claimed. While if *q* < 0.85, the combined effect is synergism; and if *q* < 0.55, significant synergism is claimed.
